# A Marseillevirus isolate from the Brazilian wetlands

**DOI:** 10.1007/s00705-025-06456-6

**Published:** 2025-11-21

**Authors:** Matheus Felipe dos Reis Rodrigues, Nidia Esther Colquehuanca Arias, Talita Bastos Machado, João Victor Rodrigues Pessoa Carvalho, João Pessoa Araújo Júnior, Luiz Henrique Rosa, Luiz Carlos Junior Alcantara, Rodrigo Araújo Lima Rodrigues, Victória Fulgêncio Queiroz, Jônatas Santos Abrahão

**Affiliations:** 1 Departamento de Microbiologia, Universidade Federal de Minas Gerais (UFMG), Belo Horizonte, Minas Gerais Brasil; 2 Department of Genetic, Microbiology, and Immunology, São Paulo State University (UNESP), Botucatu, São Paulo, Brazil; 3 Instituto Rene Rachou, Fundação Oswaldo Cruz and Expanded Navigation for Intensive and Optimized Surveillance (NAVIO) Network, 30190- 002 Belo Horizonte, Brazil; 4 Centro Tecnológico de Vacinas da Universidade Federal de Minas Gerais (CT- Vacinas/UFMG), Belo Horizonte, Minas Gerais Brasil; 5 Department of Biological Sciences, Virginia Tech, 926 West Campus Drive, Blacksburg, 24061 VA USA

**Keywords:** Marseillevirus, Pantanal, Giant viruses, Amoeba, Virus isolation

## Abstract

Marseilleviruses are giant viruses that infect amoebas and have been isolated from various environments. Here, we describe a new strain, isolated from a water sample collected from the Paraguay River in the Brazilian Pantanal, which we have named "Marseillevirus pantanense". Transmission and scanning electron microscopy revealed icosahedral particles approximately 190 nm in diameter. Genomic and phylogenetic analyses show that M. pantanense has a 379,900-bp genome with 496 open reading frames, including 12 ORFans and two tRNAs. Furthermore, phylogenetic analysis places M. pantanense within Marseillevirus lineage A, group I. The findings of in this study underscore the importance of isolating new viruses to expand our understanding of the abundance and distribution of giant viruses in Brazilian biomes.

**Supplementary Information:**

The online version contains supplementary material available at 10.1007/s00705-025-06456-6.

## Introduction

Since the discovery of Acanthamoeba polyphaga mimivirus in 2003 [1], our understanding of the virosphere has expanded significantly. The subsequent isolation of several giant viruses – characterized by particles with distinct morphologies and sizes, as well as large genomes – has contributed to a better understanding of their diversity and abundance. Among them are the marseilleviruses, which, since their first isolation in 2009 [2], have been found in a variety of sample types, predominantly those derived from freshwater sources [3–10]. Amoebas of the genus *Acanthamoeba* appear to serve as environmental hosts for these viruses, given the consistent success in isolating them in the laboratory using this host as a platform [2, 3, 7, 8].

Morphologically, marseilleviruses have icosahedral particles with diameters ranging from 180 to 250 nm [2, 11], and all known isolates are classified within the phylum *Nucleocytoviricota*, order *Pimascovirales*, and family *Marseilleviridae* [12]. Marseillevirus genomes are circular, double-stranded (ds) DNA molecules. Among the isolated marseilleviruses, genome sizes range from 348 to 404 kbp, with a GC content of 42.9% to 45.2%, and they have between 386 and 515 predicted genes [10, 11]. Most of these genes are ORFans (genes that have no homologs in public databases) or encode uncharacterized proteins (proteins that have homologs in public databases but whose function is not known), which is a common feature of giant viruses [2, 13]. However, although rare, some marseilleviruses encode translation-related factors [14], including tRNAs and aminoacyl-tRNA synthetases (aaRSs), as observed in Tokyovirus, Marseillevirus cajuinensis, and in metagenomic sequences from Loki's Castle [8, 10, 15].

Since 2011, our group has conducted numerous surveys of samples from Brazilian territory to isolate amoeba-infecting viruses. The Brazilian Pantanal, for example, is the largest continuous floodplain on the planet [16] and is therefore characterized by unique ecological features, hosting remarkable biodiversity and species richness. Here, we describe a new Marseillevirus isolate, named Marseillevirus pantanense, obtained from a water sample collected in the Brazilian Pantanal.

## Materials and methods

### Sample collection and virus isolation and purification

Water samples were collected in 2023 from the Paraguay River and surrounding areas in Mato Grosso do Sul State, Brazil, in partnership with the Expanded Navigation Project for Intensive and Optimized Surveillance (NAVIO). Using 15-mL conical tubes, 10 mL of water was collected from various locations along the river and subsequently aliquoted into microtubes.

Virus prospection was performed by inoculating crude and 1:10 diluted water samples (in phosphate-buffered saline (PBS)), into 96-well plates (Kasvi, Brazil) containing 4 × 10^4^ *Acanthamoeba castellanii* (ATCC 30010) cells in peptone-yeast extract-glucose (PYG) medium. The medium was supplemented with penicillin (100 U/mL; Cellofarm, Brazil), streptomycin (100 µg/mL; Sigma-Aldrich, USA), amphotericin B (0.25 µg/mL; Cultilab, Brazil), ciprofloxacin (0.004 mg/mL; Sigma-Aldrich, USA), vancomycin (0.004 mg/mL; Inlab, Brazil), and doxycycline (0.020 mg/mL; Sigma-Aldrich, USA). The inoculated amoebae were monitored for cytopathic effects over seven days, with three sequential passages [17]. Samples were collected from wells in which the cells exhibited a cytopathic effect and analyzed by transmission electron microscopy (TEM).

For virus purification, 1 × 10^7^ *Acanthamoeba castellanii* cells in 10 T-175 flasks containing PYG medium supplemented with penicillin (100 U/mL; Cellofarm, Brazil), streptomycin (100 µg/mL; Sigma-Aldrich, USA), and amphotericin B (0.25 µg/mL; Cultilab, Brazil) were inoculated with the virus at a multiplicity of infection (MOI) of 0.1. The cells were incubated at 30 °C, and after a cytopathic effect was observed, the supernatants were collected and ultracentrifuged twice at 13,682 × *g* through a 20% sucrose cushion for 1 hour at 5°C. The resulting pellet containing purified viral particles was resuspended in 800 µL of PBS, and viral titers were determined using the endpoint dilution method described by Reed and Muench [18].

### Transmission electron microscopy (TEM)

For TEM analysis, 7 × 10^6^ *Acanthamoeba castellanii* cells were infected with the viral isolate at an MOI of 0.1 in T-175 flasks. Once a cytopathic effect was observed, the contents of the flasks were centrifuged for 10 minutes at 1308 × *g* (Sorvall RT6000B). The pellet was then washed with 0.1 M sodium phosphate buffer (pH 7.4) and fixed with 2.5% glutaraldehyde in 0.1 M sodium phosphate buffer for at least 2 hours at room temperature with homogenization (Benfer BHS 300). The pellet was then washed again with 0.1 M sodium phosphate buffer and resuspended in the same buffer.

Subsequently, samples were fixed for 2 hours in 2% osmium tetroxide, incubated overnight in 2% uranyl acetate at 2–8°C, dehydrated through a graded ethanol series, treated with acetone, and embedded in EPON or Spurr resin. Ultrathin sections were analyzed using a Tecnai G2-12 FEI Spirit Biotwin 120 kV electron microscope at the Microscopy Center of the Universidade Federal de Minas Gerais (CM-UFMG) [10, 17].

### Scanning electron microscopy (SEM)

For SEM analysis, 1 × 10^6^ *Acanthamoeba castellanii* cells infected with the viral isolate at an MOI of 10 were used. Once a cytopathic effect was observed, the cells were centrifuged. The resulting pellet was applied to round glass coverslips coated with poly-L-lysine and fixed with 2.5% glutaraldehyde in 0.1 M cacodylate buffer for at least 2 hours at room temperature. The samples were washed three times with 0.1 M cacodylate buffer and post-fixed with 1.0% osmium tetroxide for 1 hour at room temperature. After this second fixation, the samples were washed three more times with 0.1 M cacodylate buffer and immersed in 0.1% tannic acid for 20 minutes. After a final wash with cacodylate buffer, samples were dehydrated through a graded ethanol series (35–100%). Critical-point drying using CO_2_ was then performed, followed by mounting on stubs and coating with a 5-nm gold layer. Imaging was conducted using a FEI Quanta 200 electron microscope (Thermo Fisher) at CM-UFMG.

### Immunofluorescence microscopy (IF)

For immunofluorescence (IF) analysis, 1 × 10^6^ *Acanthamoeba castellanii* cells were infected with the viral isolate at an MOI of 10. At 0, 1, 3, 6, and 8 hours postinfection (hpi), cells were harvested by centrifugation. The resulting pellets were resuspended in PBS, spread onto glass microscope slides, and fixed with methanol at −20°C. After drying, five slides were stained with whole-particle anti-MsV polyclonal antibodies produced in mice (diluted 1:400 in PBS with 0.05% Tween-20 and 3% bovine serum albumin [BSA]) for 1 hour at 37°C. Samples were subsequently incubated with anti-mouse secondary antibodies (also diluted 1:400 in PBS with 0.05% Tween-20 and 3% BSA) and Evans blue (Sigma; 0.5 g/L) for 1 hour at room temperature. Fluorescently labeled particles were visualized using a Zeiss ApoTome Axio Imager Z2 microscope with Apotome 2. Image processing was performed using Zen Lite software (Zeiss Microscopy) at CM-UFMG.

### Sequencing, genome assembly, gene prediction, annotation, and functional categorization

The genome of the purified virus was extracted using a MagMAX CORE Nucleic Acid Purification Kit and sequenced on an Illumina MiSeq platform with a single-end library prepared using an Illumina DNA Prep kit (Illumina Inc., San Diego, CA, USA). Genome assembly was performed using SPAdes (version 3.15.5) with default parameters [19, 20]. The resulting scaffold was compared to sequences in the National Center for Biotechnology Information (NCBI) database using BLASTn. Open reading frames (ORFs) were predicted using Prodigal (version 2.6.3) [21]. tRNA coding sequences were identified using tRNAscan-SE (version 2.0) [22] with default parameters and ARAGORN [23]. ORF annotation was carried out using a BLASTp search of the NCBI database, HHpred (searching PDB_mmCIF70_Mar, Pfam-A_V37, SCOPe70_2.08, and UniProt-SwissProt-viral70_3_Nov_2021 databases), and InterProScan. Functional categorization of the predicted proteins was performed based on orthologous groups of nucleo-cytoplasmic viruses (NCVOGs) [24, 25].

### Synteny and phylogenetic analysis

To perform synteny analysis, genome sequences of different Marseillevirus isolates were obtained from the NCBI GenBank database. Only Marseillevirus genomes that were isolated (excluding those reconstructed from metagenomic data) and that were complete and available in GenBank as of March 2025 were selected [10]. Because these viruses have circular genomes, the sequences were edited manually to start at the major capsid protein (MCP) gene to facilitate interpretation of the results [10]. Following sequence selection, synteny analysis was performed using the MAUVE program (version 20150226) with default parameters [26]. The genomic sequences from GenBank that were used in the analysis included Marseillevirus cajuinensis (OR991738.1), Melbournevirus (NC025412.1), Lausannevirus (HQ113105.1), Tunisvirus (KF483846.1), Brazilian Marseillevirus (KT752522.1), and Golden Marseillevirus (KT835053.1). A database of DNA polymerase delta protein sequences was used for alignment with the MUSCLE algorithm (version 5.1) [27]. Phylogenetic trees were constructed by the maximum-likelihood method using IQ-TREE software (version 1.6.12) with 1,000 bootstrap replicates to assess branch support [28, 29]. The best-fit evolutionary model was selected using ModelFinder, implemented within IQ-TREE. The resulting phylogenetic trees were visualized and edited using iTOL software (version 6) [30]. Finally, Proksee software [31] was used to produce a schematic representation of circular genome of the isolated virus, including predicted open reading frames and GC content.

## Results

**Fig. 1:**
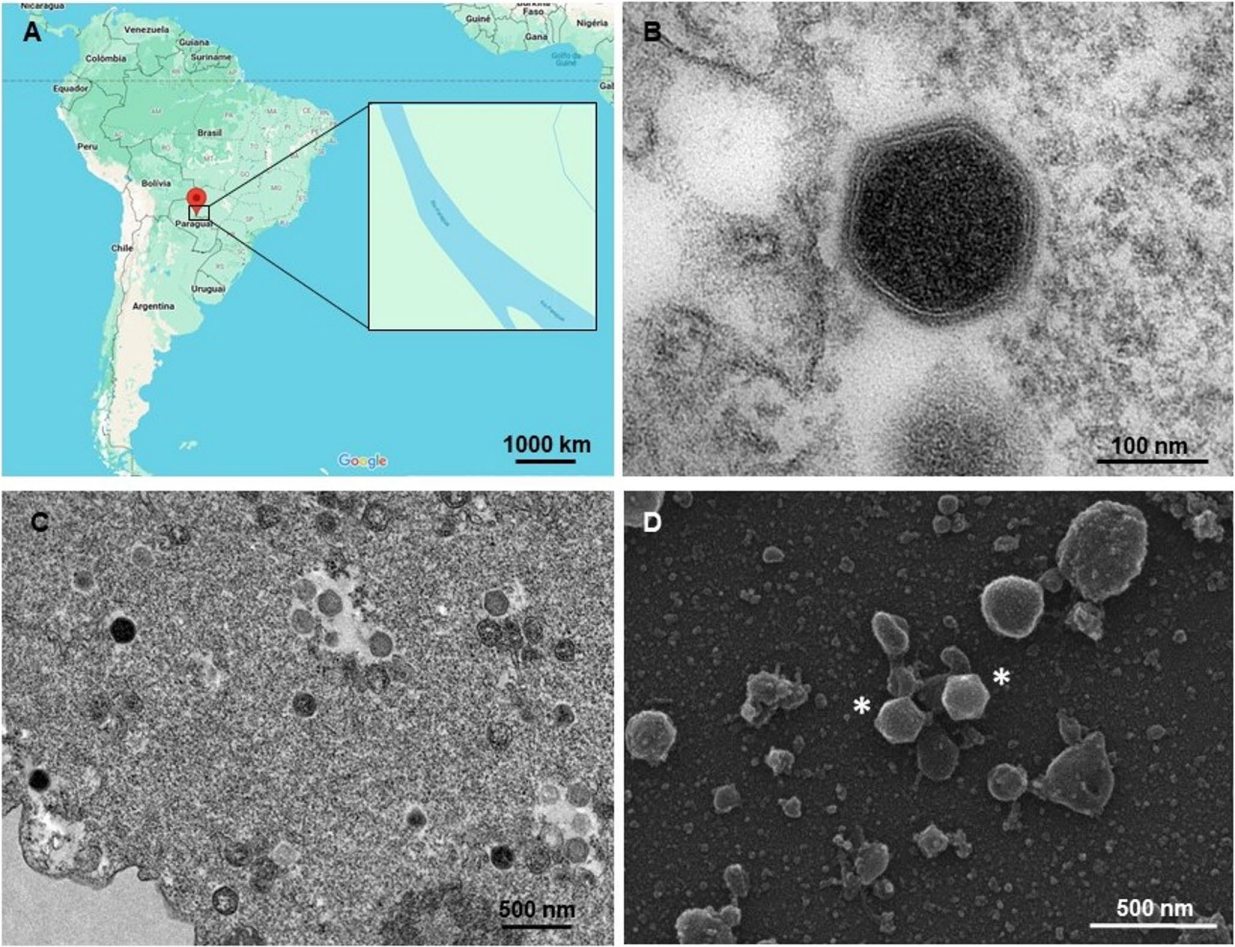
Isolation of a new marseillevirus from the Pantanal region of Brazil. **A** Map showing the location of the Paraguay River in Brazil, where sample collection was performed. **B** TEM image showing an electron-dense icosahedral virus particle. **C** TEM image of a viral factory within the cytoplasm of *Acanthamoeba castellanii*. Numerous virus particles at different stages of maturation are visible. **D** SEM image of purified icosahedral Marseillevirus particles, approximately 190 nm in diameter (indicated by asterisks)

During a 2023 expedition conducted in partnership with the NAVIO Project and the Brazilian Navy in the Brazilian Pantanal, 978 samples were collected from the Paraguay River in the communities of Porto da Manga, Porto Morrinho, Porto Esperança, Forte Coimbra, and Porto Murtinho (Fig. 1A). These samples were used to inoculate *Acanthamoeba castellanii* cells, and after three consecutive blind passages, a cytopathic effect characterized by cell rounding and lysis was observed in one sample collected from the city of Porto Murtinho, Mato Grosso do Sul, suggesting the presence of a virus.

For confirmation, the sample was prepared for TEM and SEM. The images revealed icosahedral particles approximately 190 nm in diameter, similar to those of members of the family *Marseilleviridae* (Fig. 1B and D). TEM images also revealed a viral factory (VF) occupying the periphery of the infected cell, which had an electron-lucent appearance, and some electron-dense particles representing possibly mature virus particles were also observed (Fig. 1C). Although only one sample tested positive for Marseillevirus, other giant viruses were also isolated from these samples, including a mimivirus (with and without a virophage), a moumouvirus, and a Pandoravirus.

**Fig. 2:**
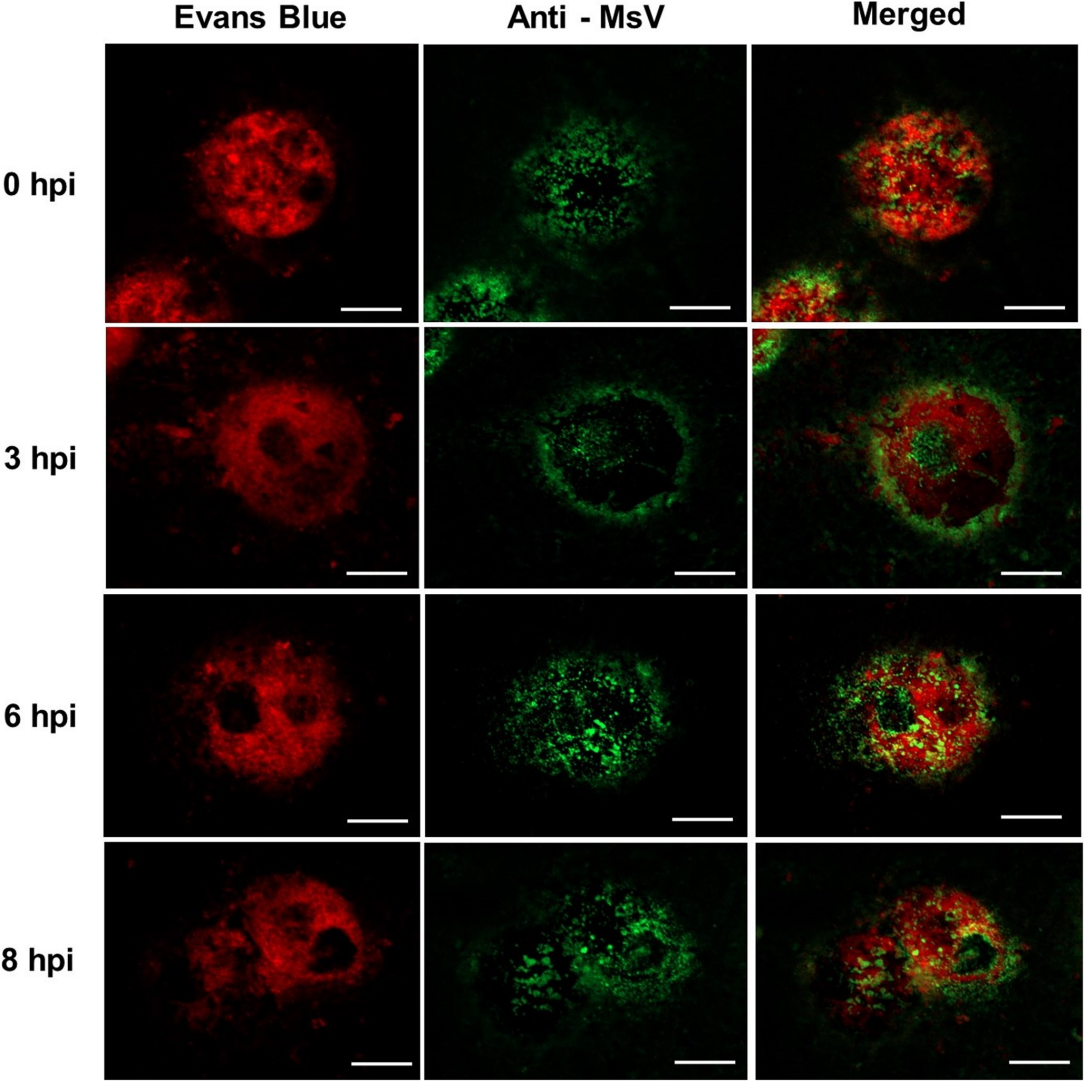
Viral replication cycle in *Acanthamoeba castellanii* assessed by IF and confocal microscopy (MOI of 10). Scale bar: 10 µm. Viral structural proteins (green) were detected using mouse polyclonal antibodies and anti-mouse IgG secondary antibodies conjugated with Alexa Fluor 488. Amoebae were counterstained with Evans blue (red)

*Acanthamoeba castellanii* was inoculated with the viral isolate at an MOI of 10 for analysis of the replication cycle by IF microscopy using anti-Marseillevirus (MsV) polyclonal antibodies and Evans blue (Fig. 2). Thirty minutes after infection (considered 0 hours postinfection [hpi] in our analyses), viral particles were observed on the surface of the amoebae, suggesting that they were still in the adsorption stage. At this time point, particles were also detected within vacuoles, indicating viral entry into the host cells. By 3 hpi, viral proteins were localized within a large vacuolar area, predominantly at the periphery of the infected cell. At 6 hpi, viral structural proteins appeared dispersed throughout the cell, forming clusters in the cytoplasm. This observation may indicate that newly formed viral particles were clustering inside vesicles, as described previously [32]. By 8 hpi, the amoebae had been lysed, releasing viral particles.

**Fig. 3:**
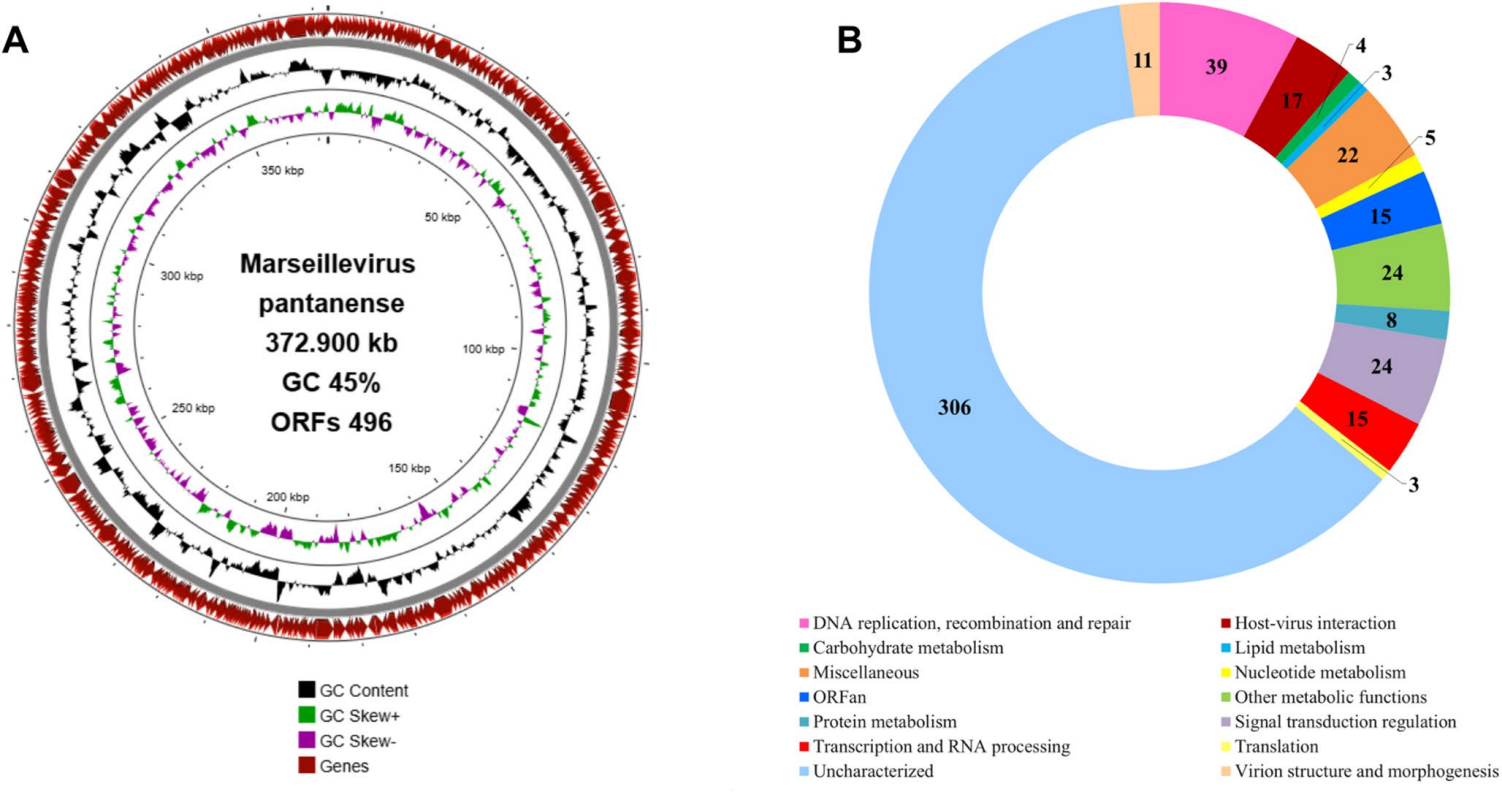
Genomic analysis of Marseillevirus pantanense. **A** Circular genome map of M. pantanense. **B** Functional categorization of the 496 predicted proteins encoded by M. pantanense

Complementing the morphological analysis, the viral isolate was further characterized through genome sequencing. Next-generation sequencing generated 1,178,375 reads. Of the 637 contigs assembled, only one contig with appropriate length and coverage matched the Marseillevirus genome, comprising 372,900 bp with 188× coverage (Fig. 3A). The provisional GenBank accession number is 2975624.

Comparison of this genome sequence with sequences in the NCBI database revealed an average nucleotide identity of 98% with the isolate Marseillevirus cajuinensis, confirming that the isolate belongs to the family *Marseilleviridae*, and we named this virus "Marseillevirus pantanense". The circular double-stranded DNA (dsDNA) genome of M. pantanense has a GC content of 45% and contains 496 predicted open reading frames (ORFs) encoding proteins (Fig. 3A). No tRNAs were detected using tRNAscan-SE; however, two tRNA sequences were identified using ARAGORN – a tRNA-Glu (tcc), with an intron of 785 bp, and a tRNA-Gln (gtc), with an intron of 1,379 bp.

Gene annotation indicated that 56 of the 496 predicted proteins are associated with DNA replication, recombination, and repair (Fig. 3B). This category includes DNA polymerase, which is commonly used as a phylogenetic marker for giant viruses, helicases, DNA ligases, various nucleases (HNH and restriction endocuclease), histones, a chaperone, DNA topoisomerase, and methyltransferases, among others. The genome also encodes proteins involved in diverse metabolic processes such as transcription and RNA processing, as well as lipases, and proteases (Fig. 3B). Within the category "virion structure and morphogenesis" are the major capsid protein (MCP) and DNA-packaging ATPase, both of which are essential for formation of viral progeny.

Most of the proteins (212; 42.7%) of M. pantanense were classified as uncharacterized (Fig. 3B). Additionally, 12 proteins were identified as ORFans, as they showed no significant similarity to any sequences in the databases used for gene annotation (BLASTp, HHpred, and InterProScan). The ORFans ranged in size from 132 to 651 amino acids.

Using the complete genome sequence of M. pantanense and those of M. cajuinensis, Melbournevirus, Lausannevirus, Tunisvirus, Brazilian Marseillevirus, and golden Marseillevirus, which were obtained from the GenBank database, collinearity among these sequences was analyzed using MAUVE software. Since the genomes of *Marseilleviridae* representatives are circular, the gene encoding the major capsid protein (MCP) was used as the starting point to align all sequences consistently.

**Fig. 4:**
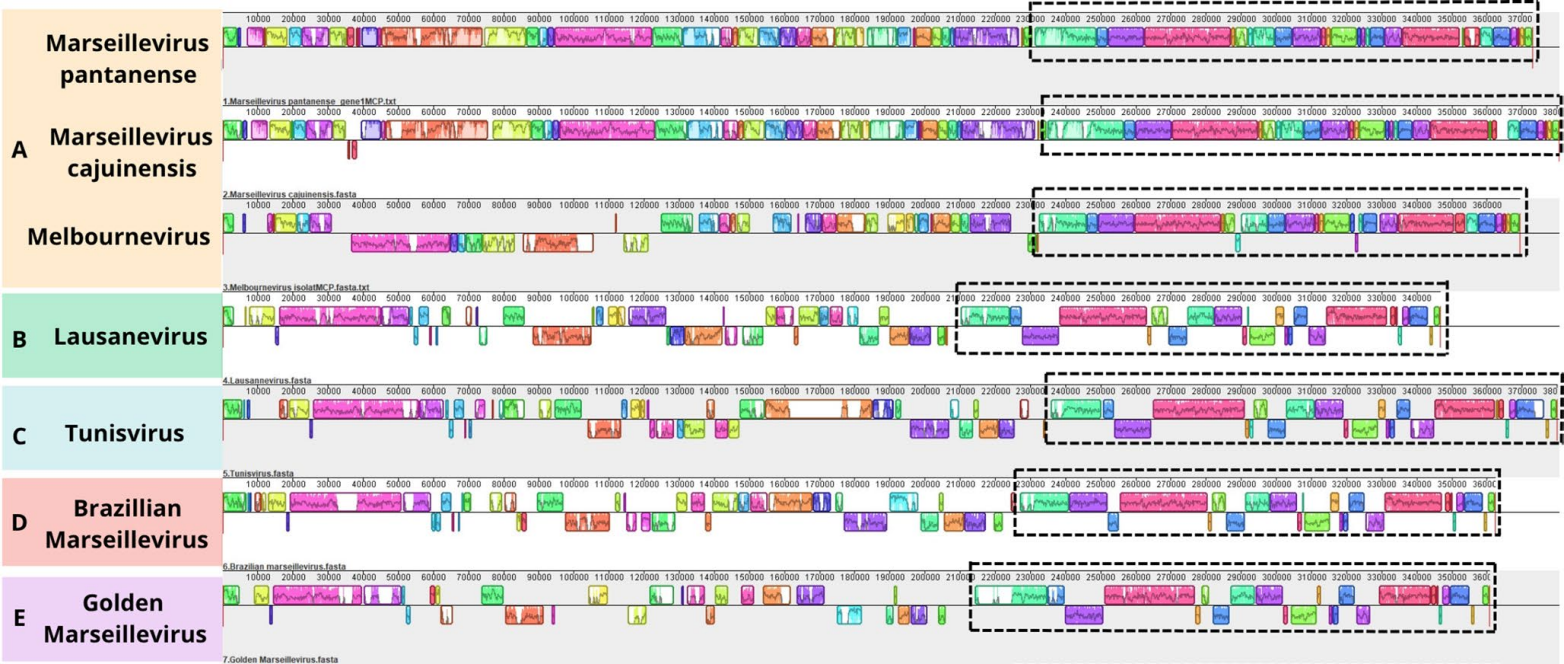
Gene synteny analysis of members of the family *Marseilleviridae* representing the five described phylogenetic lineages and Marseillevirus pantanense. Each horizontal line corresponds to the genome sequence of a different virus, identified on the left. The letters A, B, C, D, and E denote the respective phylogenetic lineages of each virus. Colored blocks indicate homologous regions shared between sequences, while uncolored areas represent unique regions exclusive to a given virus. Note: Due to their circular genomes, sequences were aligned starting from the major capsid protein (MCP) gene. Dashed rectangles indicate a conserved region previously described in Marseillevirus genomes

The overall synteny of the gene blocks in M. pantanense was found to be similar to that of M. cajuinensis and Melbournevirus (Fig. 4). Based on this similarity, we infer that M. pantanense belongs to lineage A, group I [10], whereas Lausannevirus, Tunisvirus, Brazilian Marseillevirus, and golden Marseillevirus represent lineages B, C, D, and E, respectively [10]. This analysis also revealed a conserved genomic region (indicated by dashed rectangles) that was described previously in Marseillevirus genomes [33].

**Fig. 5:**
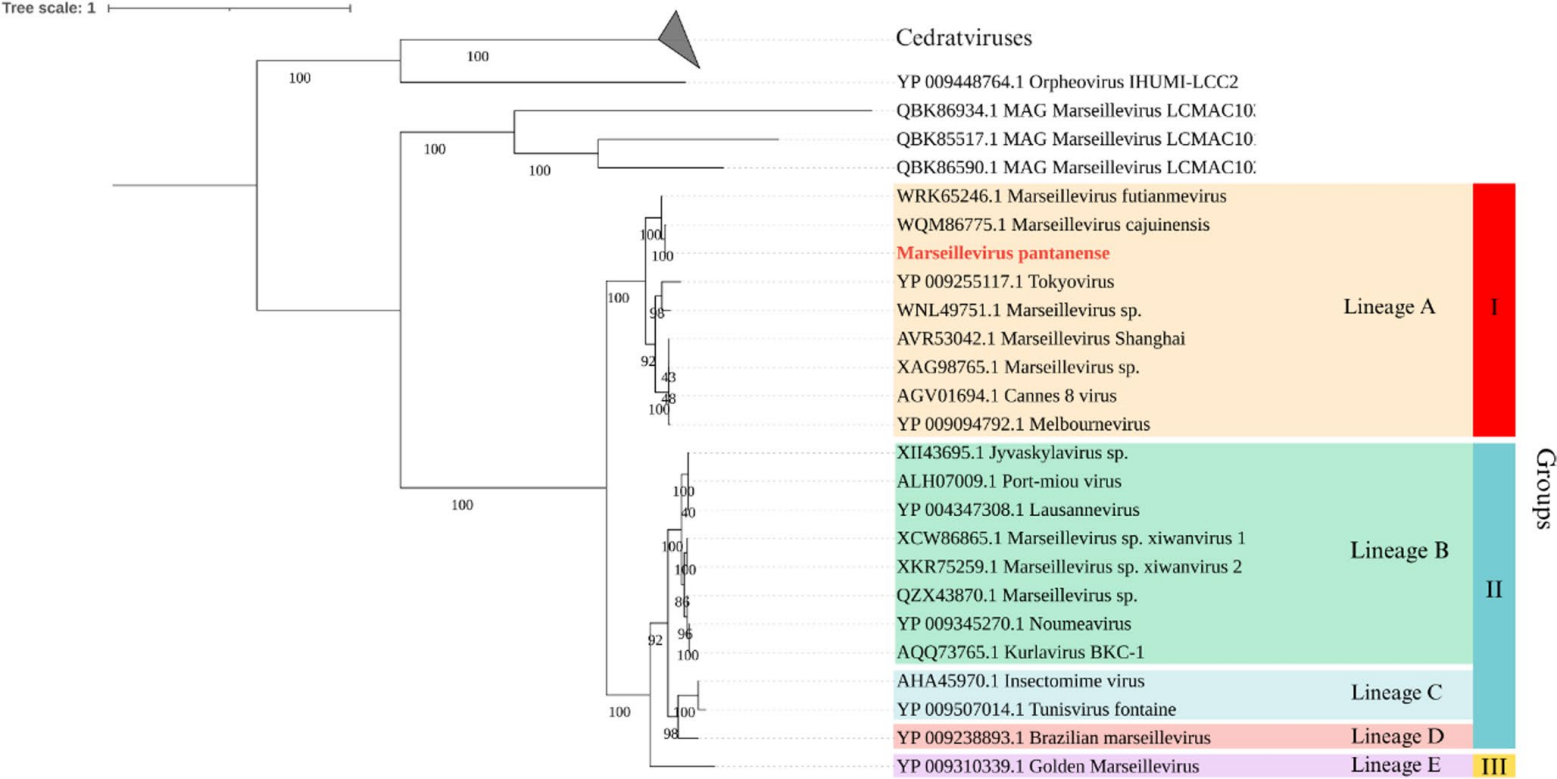
Phylogenetic tree of members of the family *Marseilleviridae* based on DNA polymerase sequences. The tree was constructed using the maximum-likelihood method with 1000 bootstrap replicates for statistical support. The best-fit model, selected by ModelFinder in IQ-TREE, was LG + F + I + G4. The tree was rooted using cedratviruses and Orpheovirus as outgroups

Phylogenetic analysis was performed with other members of the family *Marseilleviridae* to determine the relationship of M. pantanense within the group. DNA polymerase sequences were used to construct a phylogenetic tree that included the new isolate and other giant virus sequences available in the GenBank database (Fig. 5). This analysis showed that M. pantanense clustered within lineage A, group I.

## Discussion

Here, we describe an isolate of a new giant virus infecting amoebae, which we have named "Marseillevirus pantanense". This virus was isolated from samples collected from the Paraguay River and surrounding riverside communities in the Pantanal biome of Mato Grosso do Sul state. Like most isolates of the family *Marseilleviridae*, M. pantanense was obtained from freshwater environments [2–9], likely reflecting the presence of its protist host in these habitats.

TEM and SEM revealed the presence of icosahedral particles approximately 190 nm in diameter, along with electron-lucent viral factories, consistent with observations reported for other *Marseilleviridae* members [9, 11]. Although IF analysis confirmed the previously described steps of the Marseillevirus replication cycle, to the best of our knowledge, this is the first report of localization or migration of structural proteins to the cell periphery at 3 hpi. While the mechanisms underlying this phenomenon remain unclear, we hypothesize that compartmentalized production of viral structural components at the cell periphery may optimize their subsequent assembly within viral factories [9, 11]. Alternatively, some viral particles remaining in the 3 hpi supernatant might still have been in the process of entering cells.

Analysis of the M. pantanense genome revealed features consistent with other *Marseilleviridae* members [2, 3, 5, 7, 11], including genome size (372.9 kbp), number of predicted genes (496), and GC content (45%). Like Tokyovirus and M. cajuinensis, the M. pantanense genome contains predicted tRNA genes, specifically tRNA-Glu and tRNA-Gln. Recently, five other Marseillevirus genomes (Marseillevirus marseillevirus, Melbournevirus, Insectomime virus, Tunisvirus, and golden marseillevirus) were also predicted to encode tRNAs [10]. Future experimental and transcriptomic analyses are needed to confirm the presence of tRNA genes in Marseillevirus genomes.

Functional annotation showed that a large proportion of the M. pantanense proteome (212 proteins) consists of uncharacterized or hypothetical proteins, which aligns with findings for other giant viruses infecting amoebae [13]. Further studies are needed to elucidate the functions of these abundant unknown proteins, which are commonly found in nucleocytoplasmic large DNA viruses (NCLDVs) [13]. As observed in the functional annotation of other Marseillevirus genomes, M. pantanense encodes restriction endonucleases (15 in total), which may have been acquired through horizontal gene transfer [2] and contribute to the genomic mosaicism of Marseillevirus. M. pantanense encodes a methylase, which we hypothesize is involved in the inhibition of host transcripts to favor the transcription of early, intermediate, and late viral factors.

Finally, genomic and phylogenetic analyses placed M. pantanense within lineage A, group I of the family *Marseilleviridae* [10]. This work contributes to a broader understanding of the distribution and diversity of marseilleviruses in environments with unique biotic and abiotic characteristics, such as the Brazilian Pantanal.

## Electronic Supplementary Material

Below is the link to the electronic supplementary material.

ESM 1 XLSX (33.9 KB)

ESM 2 TXT (376 KB)

ESM 3 FASTQ (394 MB)

## Data Availability

The genome sequence of Marseillevirus pantanense will be publicly available upon the publication of the article. The raw and analyzed genome data can be accessed in the supplementary materials.
